# Efficacy of Tocilizumab in COVID-19: Single-Center Experience

**DOI:** 10.1155/2021/1934685

**Published:** 2021-12-30

**Authors:** Safak Kaya, Seyhmus Kavak

**Affiliations:** ^1^Department of Infectious Diseases, Gazi Yaşargil Training and Research Hospital, University of Health Sciences, Diyarbakir, Turkey; ^2^Department of Radiology, Gazi Yaşargil Training and Research Hospital, University of Health Sciences, Diyarbakir, Turkey

## Abstract

**Background:**

Cytokine release syndrome can be observed during the course of COVID-19. Tocilizumab is used for treating this highly fatal syndrome. We think that the starting time of tocilizumab is important. In this article, we aimed to discuss the efficacy of tocilizumab and to review the necessity of starting it in the early period and the laboratory values that guide us in determining the time of this early period.

**Methods:**

This retrospective study includes a total of 308 patients with a diagnosis of COVID-19 who were treated with tocilizumab, who were hospitalized in the University of Health Sciences, Gazi Yaşargil Training and Research Hospital between July 2020 and December 2020. The data of the patients were recorded on the day of hospitalization, the day of taking tocilizumab (day 0), and the 1st day, 3rd day, 7th day, and 14th day after taking tocilizumab. Data included age, gender, underlying diseases, where the patient was followed, duration of symptoms before admission to the hospital, duration of oxygen demand before tocilizumab, fever, saturation, and laboratory values. Patients were divided into the mortality group (group 1) and the survival group (group 2), and all data were compared.

**Results:**

The study consisted of 308 COVID-19 patients divided into two groups: the mortality group (group 1, *n* = 135) and the survival group (group 2, *n* = 173). The median age of the patients was 60 (min–max: 50-70) years, 75.3% (*n* = 232) were male, and 56.8% had at least one comorbidity. While 88.9% of group 1 was in the intensive care unit, 26.6% of group 2 received tocilizumab while in the intensive care unit, and there was a statistically significant difference. Median SpO_2_ values and lymphocyte counts were significantly lower in group 1 than in group 2, both on the day of hospitalization and on the day of the first dose of tocilizumab treatment (*p* < 0.001 for both). C-reactive protein, d-dimer, and alanine aminotransferase values were higher in the mortal group on the first day of hospitalization, and this was significant (*p* = 0.021, *p* = 0.001, and *p* = 0.036, respectively). In our study, d-dimer was 766.5 ng/mL in the survivor group and 988.5 ng/mL in the mortal group. In our patient group, the mean lymphocyte count was 700 × 10^3^/mm^3^ in the group that survived the first day of TCZ and 500 × 10^3^/mm^3^ in the mortal group. In addition, the CRP value was 135.5 mg/L in the survivor group and 169 mg/L in the mortal group. There was no difference between ferritin values.

**Conclusions:**

Tocilizumab is still among the COVID-19 treatment options and appears to be effective. But the start time is important. In order to increase its effectiveness, it may be important to know a cut-off value of the laboratory findings required for the diagnosis of cytokine release syndrome. Further studies are needed for this.

## 1. Background

At the end of 2019, a new virus causing pneumonia cases was detected in Wuhan, China, and then, this virus has spread rapidly and became a pandemic affecting almost all countries around the world. World Health Organization (WHO) announced “COVID-19” as the name of this new disease on February 2020, and later, the name of the causative virus was declared as severe acute respiratory syndrome coronavirus 2 (SARS-CoV-2) [1]. Despite the increasing number of cases, there is still no approved specific treatment for the COVID-19 disease today. Therefore, seeking significant treatment options that can be used in the fight against the COVID-19 disease is crucial. High levels of IL-6 detected in patients hospitalized for severe COVID-19 disease have demonstrated that a dysfunctional immune and inflammatory response may occur in the pathogenesis of severe disease. In such cases, patients exhibit a hyperinflammatory state similar to cytokine release syndrome [2, 3].

Patients with cytokine release syndrome may show rapid disease progression and may be lost because of cardiovascular collapse and multiple organ damage. Therefore, early recognition and treatment of the cytokine release syndrome is of critical importance for these patients [4]. Interleukin- (IL-) 6 is a potent proinflammatory mediator that is important in immune defense and immune-mediated disease. IL-6 is considered to be the most important mediator in the cytokine release syndrome [5]. Tocilizumab (TCZ) is a recombinant humanized anti-IL-6 receptor (IL-6R) monoclonal antibody blocker. Tocilizumab inhibits the binding of IL-6 to its receptors while competing with both soluble and membrane-bound forms of the human IL-6 receptor (IL-6R), thus reducing the proinflammatory activity of this cytokine [6].

Although there are data showing that TCZ decreases the severity of the disease in the cytokine release syndrome, which can be observed in the course of COVID-19 disease and is associated with mortality, there are other studies showing the opposite [7–10]. There are publications showing that TCZ is not effective in preventing death in patients with moderate COVID-19 [11, 12]. There are also publications that found limited clinical improvement in patients receiving TCZ in severe COVID-19 and that clinical studies are needed to further evaluate the benefit of TCZ [13].

In this study, we aimed to present the real-life data of 308 patients for whom TCZ was given for the cytokine release syndrome and to contribute to the literature. We think that the starting time of TCZ is important. In this article, we aimed to discuss the efficacy of TCZ and to review the guiding values of laboratory findings on when this early period may occur, although it is said to be started in the early period.

## 2. Methods

This study is a retrospective study examining a total of 308 COVID-19 patients treated with TCZ who were hospitalized in the University of Health Sciences, Gazi Yaşargil Training and Research Hospital between July 2020 and December 2020.

The following criteria are considered as the indication for TCZ: above 18 years of age, diagnosed with COVID-19, SpO_2_ < 93% and oxygen requirement, increase in at least two consecutive inflammatory markers (C-reactive protein, ferritin, d-dimer) and/or elevation of liver enzymes, and cytopenia.

Tocilizumab is not recommended in patients with suspected or confirmed bacterial infection, a history of diverticular disease or gastrointestinal perforation, alanine aminotransferase (ALT) or aspartate aminotransferase (AST) > 5 times the upper limit of normal at baseline, and an absolute neutrophil count < 500 × 10/mm^3^ at the baseline. Tocilizumab was administered at a dosage of 8 mg/kg (max. 800 mg) by two consecutive intravenous infusions in an interval of 12 h. A third infusion was given if necessary, based on the clinical response 24 h after the second dose. It was diluted in 100 mL 0.9% sodium chloride solution and intravenously administered for 60 min. Data were obtained from the hospital database. The data of the patients were recorded on the day of hospitalization, the first day of TCZ (day 0), and the 1st day, 3rd day, 7th day, and 14th day. These data include demographic information such as age, sex, underlying diseases, follow-up unit, duration of symptoms before admission to the hospital, duration of oxygen requirement before TCZ, duration of symptoms, values of fever, saturation, lymphocyte, C-reactive protein (CRP), d-dimer, ferritin, ALT, and AST oxygen support system received by patients before and after TCZ [low (nasal cannula, mask with a reservoir bag) or high flow (Continuous Positive Airway Pressure, ventilator)], duration of post-TCZ oxygen support, length of hospital stay, and survival status. All patients received oral antiviral (favipiravir), 6 mg/day IV dexamethasone, and IV antibiotic treatment. The patients were divided into two groups as mortality (group 1) and survival (group 2), and the data of these two groups were compared.

The study was approved by the ethics committee of Gazi Yaşargil Training and Research Hospital, University of Health Sciences (25.09.2020 dated and 583 numbered).

### 2.1. Statistical Analysis

SPSS software ver. 23 (SPSS Inc., Chicago IL, USA) was used in performing statistical analyses. The Shapiro-Wilk normality test was used to examine for normality of distribution. Categorical variables were presented as frequencies (percentages) and compared with the chi-square test (or Fisher's exact test, where appropriate). Nonnormally distributed continuous variables were presented as median with interquartile range (IQR, 25th and 75th percentiles) and compared with the Mann–Whitney *U* test between the groups. The Wilcoxon signed rank test was used for the comparison of nonnormally distributed variables at the different time points in each group. After the possible factors identified by univariate analyses, a multivariable logistic regression model used to determine independent predictors of hospital mortality included age, presence of hypertension, diabetes mellitus, levels of serum CRP and lymphocyte counts, temperature, and SpO_2_ at the first dose of TCZ. The logistic regression model was statistically significant, *χ*^2^(4) = 130.340, *p* < 0.001. The model fit was assessed using the Hosmer and Lemeshow goodness-of-fit test. The model explained 46.2% (Nagelkerke R^2^) of the variance in predicting hospital mortality and correctly classified 79.5% of cases. For all comparisons, a value of *p* < 0.005 was considered statistically significant.

## 3. Results

The study consisted of 308 COVID-19 patients divided into two groups: the mortality group (group 1, *n* = 135) and the survival group (group 2, *n* = 173). Median age of patients was 60 (min–max: 50-70) years, 75.3% (*n* = 232) were male, and 56.8% had at least one comorbidity, of which hypertension (34.1%) and diabetes mellitus (23.4%) were the most common. There was a difference between groups 1 and 2 with regard to hypertension, diabetes mellitus, chronic renal failure, and cardiovascular diseases (*p* < 0.001, *p* < 0.001, *p* =0.046, and *p* < 0.001, respectively). Although RT-PCR was negative for SARS-CoV-2 RNA in 10% (*n* = 31) of patients, they had typical chest CT findings and clinical symptoms consistent with COVID-19. The mean time between symptom onset and hospitalization was 7 days. There was no difference between the groups. In addition, the mean time from symptom onset to TCZ administration was 11 days. The time from the onset of symptoms to the initiation of TCZ was longer in group 1. However, there was no difference between the groups. There was no difference between the two groups in terms of the duration of oxygen support before TCZ.

The duration of oxygen supplementation after TCZ was longer in group 1 (Table 1) (respectively, *p* = 0.452, *p* = 0.755, *p* = 0.133, and *p* = 0.079).

After TCZ, the day of hospitalization was significantly longer in group 1 (Table 1) (*p* = 0.001). A total of 166 patients received TCZ while in the intensive care unit. 88.9% of group 1 and 26.6% of group 2 received TCZ while in the intensive care unit, and there was a statistically significant difference (Table 1) (*p* < 0.001). There was a difference between the oxygen support systems (low and high oxygen flow support systems) needed by the patients before and after TCZ (Table 1) (*p* < 0.001). Group 1 received a higher percentage of high-flow oxygen.

The median SpO_2_ values were 84.5% in group 1 and 88.5% in group 2 on the day of hospitalization and 80% in group 1 and 84% in group 2 on the day of the first dose of TCZ treatment, and there was a statistical difference between the two groups (<0.001). On the day of hospitalization, mean temperature was 37.4°C in group 1 and 37°C in group 2, and there was a difference between the groups (*p* = 0.005). On the day of TCZ treatment, the temperature was 37.85 C in group 1 and 37.5 in group 2. There was no difference between the two groups (*p* = 0.073).

The d-dimer value was significantly different between the groups, both on the day of hospitalization (400 ɣ 260.5 ng/mL) and on the first day of TCZ administration (988.5 ɣ 776.5 ng/mL) (Table 2) (*p* < 0.001 for both). There was a significant difference in ALT levels between the two groups (Table 2). There was no difference between the groups in ferritin and AST values both on the first day of hospitalization and on the first day of TCZ.

**Figure 1:**
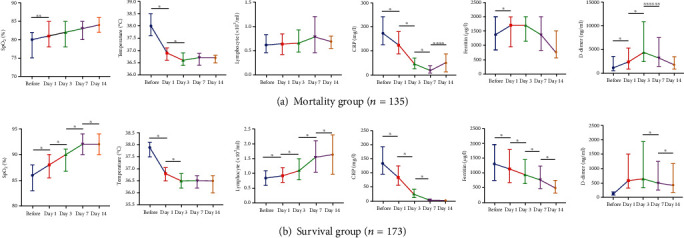
Comparison of the (a) SpO_2_, (b) temperature, (c) lymphocytes, (d) CRP, (e) ferritin, and (f) d-dimer parameters in the mortality group (a) and survival group (b) at different time points after tocilizumab administration. Data are presented with median and IQR (the 25th and 75th percentiles). ^∗^*p* < 0.001, ^∗∗^*p* = 0.005, ^∗∗∗^*p* = 0.009, ^∗∗∗∗^*p* = 0.001, ^∗∗∗∗∗^*p* = 0.003, and ^∗∗∗∗∗∗^*p* = 0.048.

As shown in Figure 1, in sequential measurements, a significant increase in SpO_2_ and a significant decrease in CRP levels and temperature were seen in both groups on the first day of TCZ treatment, while a significant increase in lymphocyte counts was seen only in group 1. In addition, there was a significant decrease in serum ferritin levels in group 2, while there was a significant increase in serum ferritin levels in group 1 (*p* < 0.001 for both) (Figure 1).

Following the first day of TCZ, a sustained increase in SpO_2_ values and lymphocyte counts was seen only in group 1 even after day 14 of treatment (Figure 1).

In group 2, 26 (21.7%) patients who required low-flow supplemental oxygen before TCZ required high-flow oxygen or ventilation support (invasive or noninvasive) after TCZ.

**Figure 2:**
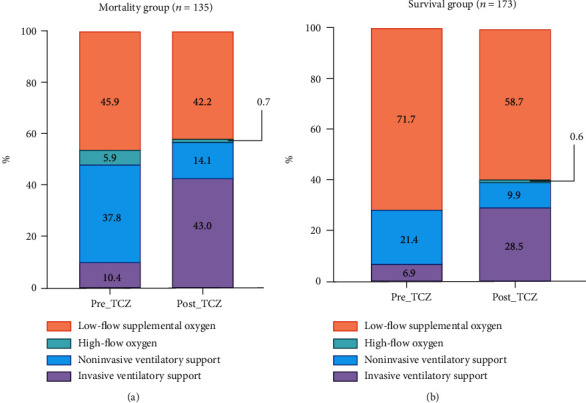
Comparison of oxygen need categories between before and after the first dose of tocilizumab in the (a) mortality group and (b) survival group.

However, 7 of 48 patients who needed high-flow oxygen or ventilation support before TCZ started to receive low-flow oxygen after TCZ. Five of the 73 patients in group 1 needed high-flow oxygen or ventilation support before TCZ, and 5 started receiving low-flow oxygen after TCZ. The ratio of oxygen categories received by patients before and after TCZ administration is shown in Figure 2.

In multivariate analysis, for one unit increase in lymphocyte count, there was a 68% reduction in hospital mortality, while one unit increase in SpO_2_ corresponds to a 17% decrease in hospital mortality. Patients with hypertension were 2.59 times more likely to die than those without, while those with diabetes mellitus were 2.96 times more likely to die than those without (Table 3).

## 4. Discussion

Although it affects the whole world, there is still no effective treatment for COVID-19. Cytokine release syndrome seen during the COVID-19 disease course increases the risk of death [14]. Approved by the Food and Drug Administration for treating cytokine release syndrome, the IL-6 receptor antibody TCZ may provide clinical benefit for selected COVID-19 patients with high inflammatory biomarkers [15]. We administered TCZ treatment in patients with cytokine release syndrome. This study includes real-life data of 308 patients who were followed up with the diagnosis of COVID-19 and received TCZ because of cytokine release syndrome. We compared the data of 173 survivors and 135 patients who died. We think that the starting time of TCZ is important. In this article, we aimed to discuss the efficacy of TCZ and to review the guiding values of laboratory findings on when this early period may occur, although it is stated that it should be started in the early period.

Note that >50% of the patients in our study group were male. Our results showed that advanced age, diabetes, hypertension, chronic obstructive pulmonary disease (COPD), coronary artery disease, and renal failure are associated with mortality.

Early initiation of TCZ therapy in patients with signs of cytokine release syndrome is important [4]. In the study by Knorr et al., the mean time from admission to receiving TCZ was 3.7 days [13]. Toniati et al. have started TCZ treatment five days after admission [16]. Xu et al. have stated that TCZ should be initiated early such that the symptoms can be controlled [17]. In the study by Vu et al., it has been reported that only 9 of 31 patients who received TCZ in the early period progressed to mechanical ventilation [14]. In our study, the time from the onset of symptoms to the onset of TCZ was shorter in the survival group and was 4.5 days, whereas this value was five days in the mortality group. Mortality was higher in patients who were followed up in the intensive care unit, receiving high-flow oxygen support and receiving TCZ because of cytokine release syndrome at this time. This supports the fact that the use of TCZ in the early period of cytokine release syndrome before the requirement for intensive care arises will decrease mortality. The length of hospital stay after receiving TCZ was longer in group 1. Patients who benefited from TCZ had a shorter hospital stay, so these patients had a shorter oxygen requirement. Oxygen saturation was lower in group 1 than in group 2 on the day TCZ was started. This finding is a warning that TCZ should be given early. In sequential measurements, a significant increase in SpO_2_ and a significant decrease in CRP levels and fever were present in both groups on the first day of TCZ treatment. However, while there was a significant decrease in serum ferritin levels in group 2, there was a significant increase in serum ferritin levels in group 1. A significant decrease in the ferritin level at follow-up may be a predictor of the response to TCZ.

In multivariate analysis, for each unit increase in lymphocyte count, there is a 68% reduction in hospital death rates, while each unit increase in SpO_2_ corresponds to a 17% decrease in hospital death rates. In other words, the improvement of lymphopenia in COVID-19 is directly proportional to recovery. In addition, hypertension and diabetes mellitus, which are among the underlying diseases, were found to be associated with mortality. Lymphopenia is one of the poor prognostic factors in COVID-19 disease. Considering the results at hospitalization in our study, lymphocyte values were significantly lower in the mortality group. Moreover, it was observed that the lymphocyte count was lower in the same group on the day of TCZ administration. High inflammatory markers have been associated with disease severity [11]. In our study, CRP, d-dimer, and ALT values were significantly higher in the mortality group on the first day of hospitalization. In both groups, lymphocyte values increased from the day TCZ was given. In the mortality group that survived longer than 14 days, it was observed that the value decreased again on the 14th day after TCZ and the lymphocyte count never exceeded 1000 × 10^3^/mm^3^. Although CRP values started to decrease from the first day of TCZ in both groups, it started to increase on the 14th day in the mortality group. Perhaps, these values can facilitate to evaluate when to start TCZ according to laboratory values. The median d-dimer level associated with mortality in the study of Knorr et al. was greater than 1 mg/L [13]. Xu et al. [17] have reported a basal d-dimer level of 0.8 mg/L, whereas in a study by Toniati et al. [16], this value was 0.5 mg/L. In our study, the d-dimer level was 766.5 ng/mL in group 2 and 988.5 ng/mL in group 1. In our patient group, the mean lymphocyte count was 700 × 10^3^/mm^3^ in the survival group and 500 × 10^3^/mm^3^ in group 1 on the first day of TCZ. Moreover, the CRP value was 135.5 mg/L in group 2 and 169 mg/L in group 1. There was no difference between ferritin values. However, it is clear that additional studies are required to obtain such a limit value because of the limitation of our retrospective observational study.

## 5. Conclusions

TCZ seems to be effective in the treatment of COVID-19 disease, which currently does not have an approved and effective treatment, particularly considering the high mortality risk of cytokine release syndrome. However, the increase in effectiveness depends on starting TCZ at the earliest stages of the disease when appropriate. It is especially important to start before the requirement for intensive care or high-flow oxygen support. In order to increase its effectiveness, it may be important to know a cut-off value of the laboratory findings required for the diagnosis of cytokine release syndrome.

## Figures and Tables

**Table 1:** Characteristics of the patients with COVID-19 infection who received tocilizumab in the mortality group (Group 1) and survival group (Group 2).

Variables	Overall (*n* = 308)	Group 1 (*n* = 135)	Group 2 (*n* = 173)	*p* value
Age (years)	60 (50-70)	70.5 (63-76)	58 (47-68.2)	<0.001^∗^
Gender (male)	232 (75.3)	98 (72.6)	134 (77.5)	0.326
Time from SO to HA (days)	7 (5-9)	7 (6-9)	7.0 (6-8)	0.452
Time from SO to TCZ infusion (days)	11 (10-13)	12 (10-13)	11.5 (10-13)	0.755
Duration of oxygen support before TCZ (days)	3 (2-5)	4.0 (2-5)	4.0 (2.7-5)	0.133
Duration of oxygen support after TCZ (days)	6 (4.5-9.5)	17.5 (16-22)	12.5 (8-16)	0.079
Duration of hospital stay after TCZ (days)	8 (7-10)	17.5 (16-22)	15 (14-17)	0.001^∗^
Any comorbidity	175 (56.8)	102 (75.6)	73 (42.2)	<0.001^∗^
Hypertension	105 (34.1)	71 (52.6)	34 (17.9)	<0.001^∗^
Diabetes mellitus	72 (23.4)	46 (34.1)	26 (15)	<0.001^∗^
Cardiovascular disease	63 (20.5)	40 (29.7)	23 (13.3)	<0.001^∗^
COPD	21 (6.8)	7 (5.2)	14 (8.1)	0.315
Asthma	18 (5.8)	10 (7.4)	8 (4.6)	0.302
Renal insufficient	9 (2.9)	7 (5.2)	2 (1.2)	0.046^∗^
Any immunosuppression	4 (1.3)	2 (1.5)	2 (1.2)	0.376
Other	7 (2.2)	6 (4.4)	1 (0.6)	0.191
Oxygen support category before TCZ				<0.001^∗^
Low-flow supplemental oxygen	186 (60.4)	62 (45.9)	124 (71.7)	
High-flow oxygen or ventilatory support (either noninvasive or invasive)	122 (39.6)	73 (54.1)	49 (28.3)	
Oxygen support category after TCZ				<0.001^∗^
Low-flow supplemental oxygen	158 (51.5)	57 (42.2)	101 (58.7)	
High-flow oxygen or ventilatory support (either noninvasive or invasive)	145 (47.2)	78 (57.8)	67 (39)	
None	4 (1.3)	0 (0)	4 (2.3)	<0.001^∗^
ICU admission	166 (53.9)	120 (88.9)	46 (26.6)	<0.001^∗^

^∗^Statistically significant. The categorical data are expressed as *n* (%), and the continuous data are expressed as median (interquartile range (IQR): 25th, 75th percentile). HA: hospital admission; SO: symptom onset; TCZ: tocilizumab; ICU: intensive care unit.

**Table 2:** Comparison of laboratory findings of the patients with COVID-19 infection at hospital admission and tocilizumab administration day in the mortality group (Group 1) and survival group (Group 2).

Variables	Overall (*n* = 308)	Group 1 (*n* = 135)	Group 2 (*n* = 173)	*p* value
At hospital admission				
SpO_2_ (%)	88 (83-91)	84.5 (80-90)	88.5 (85-92)	<0.001^∗^
Temperature (°C)	37.2 (36.8-37.8)	37.4 (36.9-38)	37 (36.77-37.52)	0.005^∗^
Lymphocytes (×10^3^/mL)	0.9 (0.6-1.2)	0.8 (0.6-0.9)	1 (0.6-1.4)	<0.001^∗^
CRP (mg/L)	105 (62-154.5)	139 (55-182)	105.5 (58.7-151.7)	0.021^∗^
Ferritin (*μ*g/dL)	759.5 (455.5- 1295)	779 (393-1380)	693.5 (401-1058.2)	0.964
d-dimer (ng/mL)	308 (192.5-472.5)	400 (264-837)	260.5 (173.7-386.5)	0.001^∗^
ALT (U/L)	31.5 (22-53.5)	26 (17-33)	27 (20-49.2)	0.036^∗^
AST (U/L)	39 (28.5-58)	37 (26-49)	35 (25.7-59.5)	0.675
At the first dose of TCZ				
SpO_2_ (%)	84 (79-88)	80 (75-84)	84 (81-86)	<0.001^∗^
Temperature (°C)	37.9 (37.6-38.2)	37.85 (37.5-38.1)	37.8 (37.6-38.1)	0.073
Lymphocytes (×10^9^/L)	0.7 (0.5-1)	0.5 (0.4-0.9)	0.7 (0.5-1)	<0.001^∗^
CRP (mg/dL)	152 (11-210)	169 (123-250)	135.5 (121-184.2)	<0.001^∗^
Ferritin (mg/dL)	1330.5 (789.5-2000)	1275 (842-1800)	1145 (767.5-1635.7)	0.496
d-dimer (ng/dL)	734.5 (359.5-2191)	988.5 (542-2772)	776.5 (350.7-2169.7)	<0.001^∗^
ALT (U/L)	41.0 (24-65)	35.0 (23-65)	41 (23.0-58.2)	0.005^∗^
AST (U/L)	41.5 (29-65)	55.5 (37-80)	36.5 (24.7-60.7)	0.156

^∗^Statistically significant. The categorical data are expressed as *n* (%), and the continuous data are expressed as median (interquartile range (IQR): 25th, 75th percentile). HA: hospital admission; TCZ: tocilizumab; SpO_2_: oxygen saturation measured by pulse oximetry; CRP: C-reactive protein; ALT: alanine aminotransferase; AST: aspartate aminotransferase.

**Table 3:** Multivariable logistic regression model predicting hospital mortality in patients with COVID-19 infection received tocilizumab.

Variable	OR	95% CI	*p* value
Age	1.020	0.995-1.045	0.115
SpO_2_ (%)^#^	0.832	0.786-0.882	<0.001^∗^
Lymphocyte (10^3^/mm^3^)^#^	0.322	0.132-0.788	0.013^∗^
CRP (mg/dL)^#^	1.003	0.999-1.007	0.125
Temperature (°C)^#^	1.257	0.830-1.904	0.280
Hypertension	2.594	1.341-5.019	0.005^∗^
Diabetes mellitus	2.961	1.472-5.959	0.002

^#^At the first dose of tocilizumab. ^∗^Statistically significant. OR: odds ratio; CI: confidence interval; CRP: C-reactive protein; SpO_2_: oxygen saturation measured by pulse oximetry.

## Data Availability

The data used to support the findings of this study are available from the corresponding author upon request.
